# An Adaptive Edge-Guided Dual-Network Framework for Fast QR Code Motion Deblurring

**DOI:** 10.3390/s26154879

**Published:** 2026-08-03

**Authors:** Jianping Li, Dongyang Guo, Wenjie Li, Wei Zhao

**Affiliations:** 1 Guangdong Provincial Key Lab of Robotics and Intelligent Systems, Shenzhen Institute of Advanced Technology, Chinese Academy of Sciences, Shenzhen 518055, China; ljphit@163.com; 2 University of Chinese Academy of Sciences, Beijing 101408, China; 3 School of Nano Science and Technology, University of Science and Technology of China, Suzhou 215127, China; 15034313473@163.com; 4 School of Computer Science and Artificial Intelligence, Shenzhen University of Advanced Technology, Shenzhen 518107, China; liwj@siat.ac.cn

**Keywords:** motion deblurring, QR code motion debluring, structural image prior, adaptive dual network, lightweight image deblurring

## Abstract

Unlike natural image deblurring, which primarily emphasizes perceptual quality and pixel-level fidelity, Quick Response (QR) code deblurring must retain decoding-critical structures to guarantee successful decoding. QR codes contain regular binary module grids and functional patterns with sharp boundaries, providing a strong structural prior for restoration. However, most existing learning-based QR restoration methods capture QR-specific structural information via implicit feature learning. To address this limitation, we propose an Edge-Guided Attention Block (EGAB), which explicitly extracts multi-directional edge priors and injects them into the query–key correlations of Transformer attention. Based on EGAB, we develop an Edge-Guided Restormer (EG-Restormer) for restoring severely blurred QR codes. For mildly blurred inputs, we introduce a Lightweight and Efficient Network (LENet) that performs fast restoration with low computational overhead. We further integrate EG-Restormer and LENet into an Adaptive Dual-network (ADNet), which selects the appropriate restoration branch according to the input blur level. Extensive experiments demonstrate the effectiveness of the proposed framework. EG-Restormer boosts the decoding rate by 8.67 percentage points under GoPro-only training and achieves the highest decoding rate among the evaluated methods after QRData fine-tuning. Moreover, ADNet reduces average inference latency by 19% while maintaining comparable decoding performance. These results suggest that explicit edge prior modeling enhances the recovery of structures critical for decoding, while adaptive routing provides an effective balance between decoding accuracy and computational efficiency.

## 1. Introduction

Quick Response (QR) codes have become ubiquitous in daily life, with applications in mobile payment, logistics, product traceability, industrial automation, and human–machine interaction [1,2]. The widespread use of QR codes has heightened the demand for reliable image acquisition and decoding, especially under conditions of physical damage, poor imaging conditions, or malicious manipulation [3]. Practical QR scanning is frequently performed in dynamic environments, where cameras may be handheld, mounted on mobile robots, or embedded in resource-constrained devices. Under these acquisition conditions, captured QR code images are highly susceptible to motion blur caused by camera shake, object motion, or relative movement between the imaging device and the target scene [4,5]. Such degradation may corrupt module boundaries, distort functional patterns, and ultimately lead to decoding failure. Although substantial progress has been made in natural image deblurring, restoring motion-blurred QR codes remains a distinct and practically important challenge.

**Figure 1:**
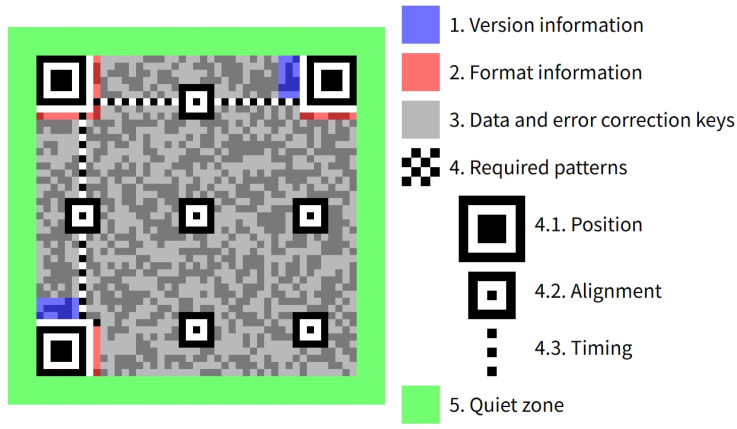
Structure of a QR code, highlighting functional elements. Source: Wikipedia [6].

As illustrated in Figure 1, a QR code is a two-dimensional matrix barcode composed of black and white modules arranged on a regular square grid, together with functional patterns such as finder markers, timing patterns, and alignment patterns [6]. These components are essential for localization, geometric correction, module sampling, and error-corrected decoding. Consequently, QR code deblurring differs fundamentally from conventional natural image restoration. For natural images, small local artifacts may remain visually acceptable, whereas minor structural errors in QR module boundaries or functional patterns may make the restored code undecodable. Therefore, QR code deblurring should not be evaluated solely in terms of perceptual quality or pixel-level fidelity. It must also preserve sharp module transitions, regular grid layouts, and structurally consistent functional patterns that support successful decoding [7,8].

Early QR code restoration methods commonly exploited task-specific structural priors through optimization-based formulations. Van Gennip et al. [9] used QR corner and finder-pattern information to constrain point spread function estimation. Rioux et al. [10] incorporated barcode symbology constraints into kernel estimation through Kullback–Leibler divergence. Sörös et al. [11] estimated blur from salient QR edges using an iterative procedure involving sharpening, blur estimation, and decoding. These studies demonstrate that QR codes contain strong structural priors that can facilitate restoration. However, such approaches generally rely on handcrafted assumptions, simplified degradation models, or iterative optimization, which may limit their effectiveness under complex or severe motion blur.

With the rapid development of deep learning, learning-based methods have become the dominant paradigm for image restoration and deblurring. Convolutional neural networks (CNNs), multi-stage architectures, generative adversarial networks (GANs), attention mechanisms, and Transformer-based models have achieved remarkable performance on natural image deblurring benchmarks [12,13,14,15,16,17]. Representative methods include progressive multi-stage restoration networks [15], U-shaped Transformer architectures [12], efficient high-resolution restoration Transformers [14], nonlinear activation-free networks [17], frequency-domain Transformers [16], and selective-frequency models [18]. These methods are effective for natural images because they can capture rich textures, long-range dependencies, and complex degradation patterns. However, directly applying them to QR code deblurring is not optimal, since they are designed primarily for perceptual or fidelity-oriented reconstruction rather than decoding-critical structural recovery.

Recent learning-based studies have addressed QR code motion deblurring, damaged-code restoration, distortion correction, and recognition using CNNs, GANs, attention mechanisms, and lightweight architectures [19,20,21,22,23,24,25]. Although these methods improve QR readability under specific degradations, most learning-based models represent QR structures mainly through implicit feature learning. By contrast, explicit QR-specific priors have been used more commonly in optimization-based restoration frameworks. Hence, an important research gap remains in integrating explicit QR structural priors into a learnable deep restoration architecture.

**Figure 2:**
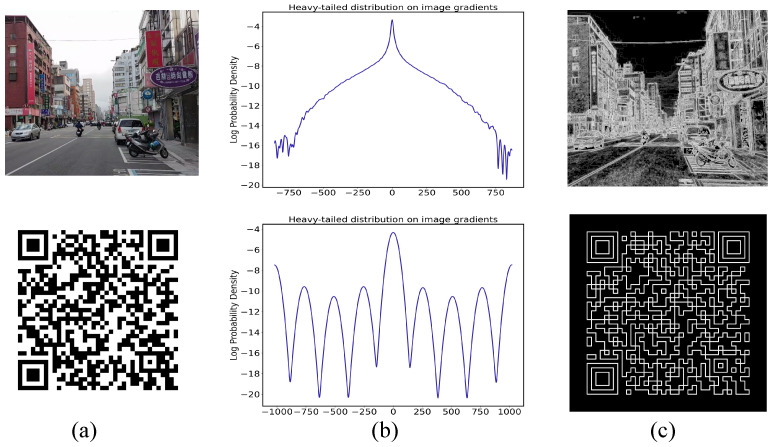
Comparison of gradient characteristics between a natural image and a QR code. The upper and lower rows show the natural-image and QR code examples, respectively. The three columns present (**a**) the original sharp images, (**b**) the log probability density of the signed vertical image gradients ($G_{y}=\partial I/\partial y$) computed using the Sobel operator, and (**c**) their gradient-magnitude maps computed as $\sqrt{G_{x}^{2}+G_{y}^{2}}$ from the horizontal and vertical Sobel gradients.

To bridge this gap, we analyze the intrinsic gradient characteristics of QR codes. As shown in Figure 2b, QR images follow a heavy-tailed distribution of signed gradients that differs from natural images because QR codes are dominated by sharp black–white transitions. Figure 2c further visualizes the corresponding gradient magnitudes, showing highly regular horizontal and vertical structures determined by the QR module grids and functional patterns. These spatially organized gradient patterns correspond directly to the structural edges of QR codes, indicating that edge information is not merely a generic low-level feature but a task-specific structural prior closely related to QR readability. Explicitly incorporating edge priors can therefore provide effective guidance when motion blur weakens module boundaries and functional patterns.

To exploit QR-specific edge priors while maintaining computational efficiency, two challenges must be addressed. First, existing learning-based QR code deblurring methods generally lack an explicit mechanism for incorporating multi-directional edge priors into feature aggregation. Consequently, they may generate visually sharp outputs while leaving local module boundaries or functional patterns structurally inconsistent, resulting in decoding failure. Second, practical QR code scanning is commonly performed on mobile, embedded, or edge devices, where computational resources and latency are strictly constrained. Applying a large restoration network to every input is inefficient for mildly blurred images, whereas relying exclusively on a lightweight model may be insufficient for severely degraded cases. Consequently, an effective QR code deblurring system should not only accurately restore severe blur but also adaptively allocate computation according to the input blur level.

To address these challenges, we propose an edge-guided adaptive framework for QR code motion deblurring. The proposed Edge-Guided Attention Block (EGAB) explicitly embeds multi-directional edge priors into Transformer attention, and the resulting EG-Restormer provides strong restoration capability for severely blurred QR codes. A lightweight LENet is introduced to efficiently process mildly blurred inputs. These two complementary branches are integrated into ADNet, which performs blur-severity-based routing and redirects decoding-failed LENet outputs to EG-Restormer, thereby balancing decoding performance and inference efficiency.

In summary, prior-driven QR restoration methods effectively exploit finder patterns, edges, intensity and gradient characteristics, and symbology constraints, but they commonly rely on handcrafted objectives, simplified degradation assumptions, or iterative optimization. Learning-based methods provide stronger representation capacity and faster inference, yet QR-specific structures are generally encoded only implicitly, and most existing approaches apply a fixed-complexity network to inputs with different blur levels. These limitations motivate the integration of explicit multi-directional QR edge priors into learnable Transformer attention and the adaptive allocation of restoration capacity according to input blur level.

The main contributions of this work are summarized as follows:

- We propose the Edge-Guided Attention Block (EGAB), which incorporates multi-directional edge priors into Transformer query–key correlations. Based on EGAB, we develop EG-Restormer, an edge-guided Transformer-based network for restoring severely blurred QR codes and preserving decoding-critical structures.
- We introduce a Lightweight and Efficient Network (LENet) for fast restoration of mildly blurred QR codes and integrate it with EG-Restormer into an Adaptive Dual-network (ADNet). Through blur-severity-based routing and decoding-failure fallback, ADNet allocates computation according to input blur level and achieves a favorable balance between decoding accuracy and inference efficiency.
- We develop an acceleration-based non-uniform motion-blur synthesis strategy for constructing a QR code deblurring dataset. Quantitative comparisons with real camera captures confirm that the proposed synthesis produces edge-spread characteristics closer to real motion blur than conventional linear-kernel synthesis. Extensive experiments further demonstrate the effectiveness of EG-Restormer and the efficiency advantages of ADNet.

## 2. Related Work

### 2.1. Structural Priors for Two-Tone Image and QR Code Restoration

Image deblurring is a highly ill-posed inverse problem, because multiple latent sharp images and blur kernels may produce the same blurred observation. Traditional methods therefore introduce image priors to constrain the solution space. Representative natural-image priors include sparse gradients, $L_{0}$ gradient regularization, total variation, dark-channel statistics, and blur-kernel constraints [5,26,27,28,29]. This perspective is closely related to classical image-segmentation techniques, in which edges, thresholds, and region information provide fundamental tools for boundary localization and the separation of homogeneous regions [30]. These concepts are particularly relevant to two-tone images, whose readability depends strongly on accurate foreground–background separation.

Two-tone images, including document text, one-dimensional barcodes, and QR codes, exhibit visual statistics that are substantially different from natural images [31]. They are usually composed of piecewise-constant foreground/background regions, a limited number of dominant intensity levels, and sparse but strong structural boundaries. These characteristics have motivated restoration methods that introduce task-specific priors rather than relying exclusively on generic natural-image statistics.

**Document and text image deblurring.** Document and text images share several properties with barcode-like patterns, such as sharp foreground-background transitions and limited intensity levels. Chen et al. [32] introduced a content-aware prior based on foreground segmentation for document image deblurring. Pan et al. [33,34] proposed $L_{0}$-regularized intensity and gradient priors for restoring blurred text, while Jiang et al. [35] formulated a two-tone prior that encourages the latent image to contain two dominant gray levels. These studies demonstrate that task-specific priors can provide stronger constraints than generic natural image priors. However, text and document images do not possess the deterministic grid layout and functional symbology of QR codes.

**One-dimensional barcode deblurring.** For one-dimensional barcodes, restoration has often been formulated as a signal recovery or statistical inference problem. Turin and Boie [36] introduced a deterministic expectation-maximization method for recovering degraded barcode signals, while Kresić-Jurić et al. [37] used hidden Markov models to infer barcode edge locations from noisy scan signals. These studies show that barcode-specific structural information can effectively constrain the deblurring process. However, one-dimensional barcodes have substantially simpler spatial dependencies than QR codes, which contain two-dimensional data modules, finder patterns, timing patterns, and alignment patterns.

**Prior-based QR code deblurring.** QR codes are particularly suitable for prior-driven restoration because their functional patterns and grid structures are explicitly defined. Choksi et al. [38] proposed an anisotropic total variation $L_{1}$ model for 2D barcodes that favors axis-aligned rectangular structures. Van Gennip et al. [9] incorporated finder-pattern information into a regularized blind deblurring framework, while Rioux et al. [10] introduced QR symbology constraints into both kernel estimation and latent-image recovery. Zheng et al. [39] exploited the intensity and gradient priors of positioning patterns for subregional blur estimation. Chen et al. [40] further proposed a fast restoration method for out-of-focus blurred QR codes using edge prior information obtained through image sensing. These studies demonstrate the effectiveness of explicit QR-specific priors for constraining the deblurring process and recovering decoding-critical boundaries.

However, most prior-based QR restoration methods rely on handcrafted degradation assumptions, iterative optimization, or a particular blur model. These constraints may limit their adaptability to complex motion blur and their compatibility with deep hierarchical feature representations. This motivates the development of learnable architectures that retain the reliability of explicit structural priors while benefiting from the representation capacity of deep neural networks.

### 2.2. Learning-Based QR Code Restoration and Correction

Learning-based methods have increasingly been applied to QR code restoration and recognition. Pu et al. [19] proposed a dual-CNN framework for restoring degraded two-dimensional barcodes. Li et al. [20] combined convolutional restoration with adaptive thresholding to handle motion blur and uneven illumination. Dong et al. [21] and Wang et al. [22] further investigated GAN-based restoration for severely motion-blurred QR codes. These studies demonstrate the potential of data-driven restoration, although QR-specific structural constraints are generally learned implicitly from the training data.

More recent studies have addressed a broader range of QR degradation and correction problems. Gu et al. [23] proposed Gs-DeblurGANv2, a lightweight QR code deblurring network in which GhostNet is used for feature extraction, a feature-pyramid architecture supports multiscale representation, and SKNet-based attention improves adaptive feature enhancement. Sirikongtham and Nimkoompai [24] used a GAN with spectral normalization to reconstruct damaged QR regions and improve scanability. Zhang et al. [25] proposed a heatmap-regression framework for locating structural control points and correcting geometrically distorted QR codes. Other systems have addressed QR recognition under uneven illumination and geometric distortion using learning-based preprocessing and deep correction models [41,42].

Although these studies all contribute to robust QR code processing, they address different degradation mechanisms and restoration objectives. Motion deblurring aims to reverse edge spreading caused by camera or object motion. Damaged-code restoration focuses on reconstructing missing or corrupted regions, whereas distortion-correction methods primarily recover geometric regularity under perspective or surface deformation. Gs-DeblurGANv2 is particularly relevant to the present study because it also considers efficient QR code motion deblurring. However, it mainly relies on implicitly learned convolutional and attention features. By contrast, the proposed EG-Restormer explicitly extracts multi-directional edge priors and integrates them into Transformer query–key correlations to strengthen decoding-critical module boundaries and functional patterns.

### 2.3. Lightweight and Practical QR Processing

Practical QR code processing is often performed on mobile phones, embedded cameras, logistics terminals, and edge devices, where latency, memory consumption, and energy efficiency are important considerations. Recent studies have investigated lightweight QR deblurring, recognition, geometric correction, and damaged-code processing for practical applications. In particular, Gs-DeblurGANv2 demonstrates that lightweight modules and multiscale feature extraction can reduce the computational burden of QR deblurring [23]. Other studies have considered damaged barcode and QR recognition in logistics environments [1], as well as lightweight visual processing in edge-based application systems [43].

These studies highlight the importance of balancing restoration performance and computational efficiency. However, most existing methods apply a single model with fixed computational complexity to all inputs, regardless of degradation severity. A powerful model may be unnecessarily expensive for mildly blurred QR codes, whereas a compact model may lack sufficient restoration capacity for severe blur. The proposed ADNet addresses this limitation through blur-severity-based routing: mildly blurred images are first processed by LENet, whereas severely blurred QR codes are assigned directly to the more powerful EG-Restormer. This design allocates computational resources according to input blur level rather than applying the same restoration network to every image.

### 2.4. Transformer-Based Image Restoration

Deep neural networks have become the dominant paradigm for image restoration and deblurring. Although convolutional neural networks provide effective local feature extraction, their spatially invariant kernels may limit the modeling of long-range dependencies under severe degradation. Transformer-based architectures address this limitation through content-dependent feature aggregation and have achieved strong performance in image restoration. Representative approaches include VIT [44], IPT [45], SwinIR [46], Uformer [12], and Restormer [14]. These methods demonstrate the effectiveness of self-attention for capturing local and nonlocal relationships in high-dimensional restoration features.

**Figure 3:**
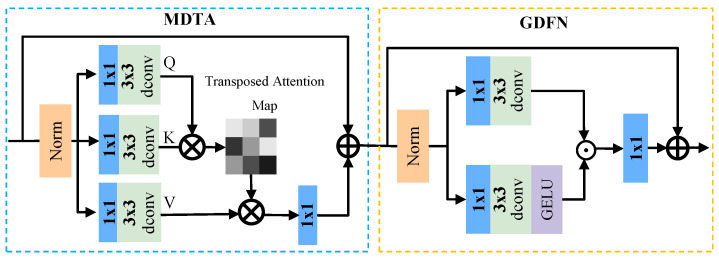
The Transformer block of the Restormer; some details are omitted for clarity. The block consists of a multi-Dconv head transposed attention (MDTA) and a Gated-Dconv feed-forward network (GDFN).

Among these architectures, Restormer provides a suitable backbone for our method because it combines a hierarchical encoder–decoder structure with computationally efficient transposed attention. Figure 3 illustrates that its Multi-Dconv Head Transposed Attention (MDTA) computes correlations across feature channels rather than all spatial positions, thereby reducing the computational cost associated with conventional global self-attention. Depth-wise convolution within MDTA and the Gated-Dconv Feed-Forward Network (GDFN) further introduces local spatial context, enabling Restormer to balance broad feature interaction with locality-aware restoration.

Nevertheless, Restormer was developed for general-purpose natural-image restoration and thus lacks explicit incorporation of QR-specific structural priors. Long-range feature aggregation alone is insufficient to guarantee the preservation of finder patterns, timing patterns, alignment patterns, or sharp module transitions required for decoding. To address this, we propose EGAB, which extends the Restormer attention mechanism by explicitly embedding multi-directional QR edge priors into query–key correlations. This design explicitly steers feature aggregation toward structural boundaries that are critical for successful decoding.

## 3. Proposed Methods

**Figure 4:**
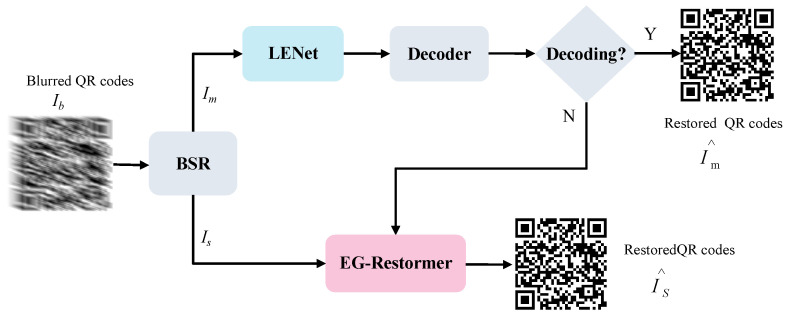
Overall architecture of the proposed Adaptive Dual-network (ADNet).

The proposed Adaptive Dual-network (ADNet) is introduced to balance restoration capability and inference efficiency for QR codes with different blur severities. As illustrated in Figure 4, ADNet consists of two complementary restoration branches: EG-Restormer for severely blurred inputs and LENet for mildly blurred inputs. A Blur Severity-based Routing (BSR) module selects the appropriate branch according to the estimated blur level, while a QR decoder provides an inference-time fallback decision for outputs restored by LENet.

**Overall Pipeline.** Given a blurred input QR code $I_{b}\in R^{H\times W\times 3}$, where H and W denote the height and width, respectively, the Blur Severity-based Routing (BSR) module first classifies $I_{b}$ into two categories according to blur level: severely blurred inputs $I_{s}$ and mildly blurred inputs $I_{m}$. Severely blurred inputs $I_{s}$ are routed to EG-Restormer model to obtain a sharpened output ${\hat{I}}_{s}$, while mildly blurred inputs $I_{m}$ are first processed by LENet for fast deblurring. The output of LENet is then evaluated for successful decoding by a decoder to determine whether successful decoding can be achieved. If decoding fails, the output is rerouted to EG-Restormer for further refinement. Otherwise, the output of LENet ${\hat{I}}_{m}$ is regarded as the final restored output of ADNet. Thus, the proposed ADNet is designed not only to improve decoding reliability but also to improve the efficiency of the deblurring model.

### 3.1. Edge-Guided Restormer (EG-Restormer)

To build an effective Transformer model for restoring severely blurred QR codes, this study explicitly leverages the unique structural properties of QR codes, particularly their sharp edges, which are essential for successful decoding. We propose EG-Restormer, a Transformer-based architecture that explicitly incorporates edge priors into multi-head self-attention through the Edge-Guided Attention Block (EGAB). We first introduce the overall architecture of EG-Restormer, followed by a detailed description of the EGAB and its core component, the Edge Feature Extractor (EFE) module.

**Figure 5:**
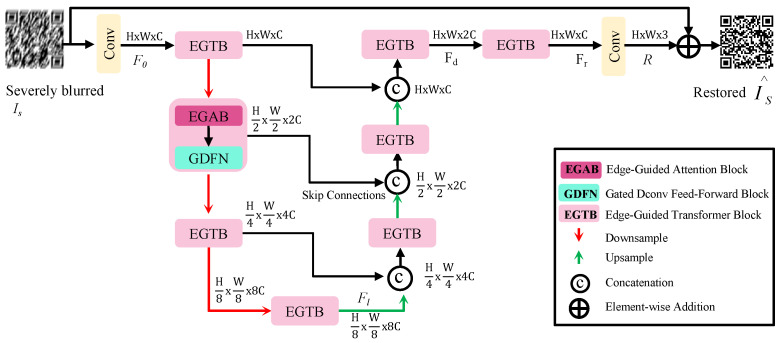
Architecture of the EG-Restormer for severely blurred QR code deblurring. EG-Restormer employs a U-shaped architecture with multiple Edge-Guided Transformer blocks (EGTB). The core component of each EGTB is the Edge-Guided Attention Block (EGAB) that incorporates an explicit edge prior into the attention block.

As shown in Figure 5, EG-Restormer adopts a U-shaped hierarchical Transformer architecture that progressively extracts and restores multiple-scale features. Given a severely blurred input QR code $I_{s}$, EG-Restormer first extracts low-level features $F_{0}$ through a convolutional layer. The extracted features are then fed into a four-layer encoder, where the spatial resolution is progressively downsampled while the channel dimension is expanded for deep feature extraction. The decoder then iteratively upscales the bottleneck features $F_{l}$ to high-resolution representations $F_{r}$. Finally, a convolutional layer is applied to generate a residual QR code *R*, which is added to the input for restoration. Both the encoder and decoder contain multiple Edge-guided Transformer Blocks (EGTBs), which guide edge-aware feature restoration and help preserve important structural details for successful decoding.

#### 3.1.1. Edge-Guided Attention Block (EGAB)

**Figure 6:**
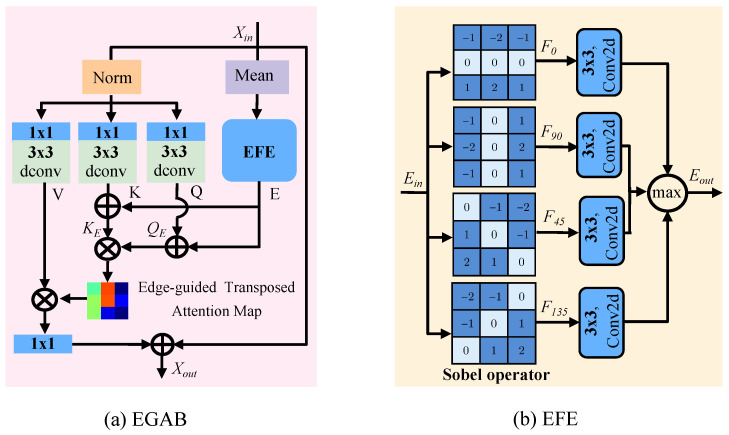
The core module of Edge-Guided Transformer Block and its components. (**a**) The Edge-Guided Attention Block (EGAB), which uses the Edge Feature Extractor (EFE) module to inject an explicit edge feature *E* into the attention mechanism. (**b**) The structure of the EFE module.

Edge sharpening is crucial for successful QR code decoding, since module boundaries, finder patterns, and timing patterns are mainly characterized by sharp black–white transitions. Conventional learning-based methods usually learn such structural information implicitly, which may lead to visually plausible but structurally unreliable restoration results. However, directly modeling such priors in conventional attention mechanisms may introduce high computational complexity. To address this issue, we propose the Edge-Guided Attention Block (EGAB), as illustrated in Figure 6a, which integrates QR code edge priors by modulating the query and key in multi-Dconv head transposed attention (MDTA) module [14].

EGAB first generates an explicit edge prior map *E* from the input feature $X_{\mathrm{in}}$ using the proposed Edge Feature Extractor (EFE), a lightweight module designed to capture multi-directional structural edges. Meanwhile, query (Q), key (K), and value (V) matrices are generated from $X_{\mathrm{in}}$ for implicit feature representation. The extracted edge prior is then used to modulate *Q* and *K*, producing edge-aware query and key features $Q_{E}$ and $K_{E}$. Finally, edge-guided transposed attention is computed based on the modulated query–key correlation. This design allows spatial edge structures, especially QR module boundaries and finder-pattern contours, to contribute more strongly to the attention calculation. The detailed formulation of EGAB is presented as follows.

Given an input feature map $X_{\mathrm{in}}\in R^{C\times H\times W}$, EGAB first generates query, key, and value features through a 1×1 convolution followed by a 3×3 depth-wise convolution:

(1) $$Q,K,V={\mathrm{DWConv}}_{3\times 3}\left({\mathrm{Conv}}_{1\times 1}\left(X_{\mathrm{in}}\right)\right),$$

where $Q,K,V\in R^{C\times H\times W}$. Meanwhile, to obtain a compact structural representation for edge extraction, channel-wise mean aggregation is applied to $X_{\mathrm{in}}$:

(2) $$X_{m}={\mathrm{Mean}}_{c}\left(X_{\mathrm{in}}\right),\;X_{m}\in R^{1\times H\times W}.$$

where ${\mathrm{Mean}}_{c}$ denotes channel-wise mean and *c* denotes the number of feature channels. Channel-wise mean aggregation provides a parameter-free and computationally efficient representation of spatial transitions shared across feature channels. It suppresses redundant or isolated channel responses while retaining dominant QR code boundaries and is less sensitive to individual high-response channels than max aggregation.

The proposed Edge Feature Extractor (EFE) is then applied to $X_{m}$ to produce an explicit edge prior map:

(3) $$E=\mathrm{EFE}\left(X_{m}\right),\;E\in R^{1\times H\times W}.$$

The edge map is used to modulate the query and key features before computing transposed attention:

(4) $$\begin{array}{cc} Q_{E} & =Q⊙(1+\alpha E),\\ K_{E} & =K⊙(1+\alpha E), \end{array}$$

where ⊙ denotes element-wise multiplication and α is a fixed edge modulation coefficient. In our implementation, α is set to 0.1. Since *E* has the shape $R^{1\times H\times W}$, it is broadcast along the channel dimension to match the shape of Q and K. In this way, spatial positions with stronger edge responses contribute more to the query–key correlation computation.

Unlike conventional edge-guided restoration methods that concatenate an edge map with image features or use edge information only as an auxiliary loss, EGAB uses the extracted edge prior to modulate the query and key representations before their correlation is computed. This enables the edge response to directly influence the attention affinity matrix rather than serving only as an additional input feature. The fixed multi-directional filters provide stable responses to the dominant horizontal, vertical, and diagonal transitions of QR modules, while the learnable projections adapt these responses to the latent feature space.

After edge modulation, $Q_{E}$, $K_{E}$, and V are reshaped into multi-head representations:

(5) $${\hat{Q}}_{E},{\hat{K}}_{E},\hat{V}\in R^{N_{h}\times C_{h}\times HW},$$

where $N_{h}$ denotes the number of attention heads and $C_{h}=C/N_{h}$ is the number of channels per head. Following MDTA, ${\hat{Q}}_{E}$ and ${\hat{K}}_{E}$ are normalized along the spatial dimension. The edge-guided transposed attention is then computed as

(6) $$A=\mathrm{Softmax}\left({\bar{Q}}_{E}{\bar{K}}_{E}^{T}\cdot T\right),$$

where $A\in R^{N_{h}\times C_{h}\times C_{h}}$ is the channel-wise attention map and $T\in R^{N_{h}\times 1\times 1}$ is a learnable temperature parameter for each attention head. Finally, the output feature is obtained by

(7) $$X_{\mathrm{out}}=X_{\mathrm{in}}+{\mathrm{Conv}}_{1\times 1}\left(A\hat{V}\right).$$

Although the attention matrix in MDTA is computed across channels rather than spatial positions, the proposed edge modulation still injects spatial structural information into the attention process. Specifically, the edge map reweights the spatial responses of *Q* and *K* before the channel-wise correlation is computed. Consequently, edge regions such as QR module boundaries and finder-pattern contours contribute more strongly to the transposed attention, encouraging the network to preserve decoding-critical structures during deblurring.

#### 3.1.2. Edge Feature Extractor (EFE)

The Edge Feature Extractor (EFE), as illustrated in Figure 6b, is designed to extract a compact and explicit structural prior from the input feature map. Specifically, EFE is designed to capture multi-directional edge features, which serve as an explicit structural prior for the subsequent attention mechanism in EGAB. Given a single-channel input feature map $E_{\mathrm{in}}\in R^{1\times H\times W}$, EFE applies four parallel Sobel operators to capture edge features along four principal orientations: $0^{\circ }$, $45^{\circ }$, $90^{\circ }$, and $135^{\circ }$. The corresponding Sobel kernels are defined as

$$K_{0^{\circ }}=\left[\begin{array}{ccc} -1 & 0 & 1\\ -2 & 0 & 2\\ -1 & 0 & 1 \end{array}\right],\;K_{45^{\circ }}=\left[\begin{array}{ccc} -2 & -1 & 0\\ -1 & 0 & 1\\ 0 & 1 & 2 \end{array}\right],\;K_{90^{\circ }}=\left[\begin{array}{ccc} -1 & -2 & -1\\ 0 & 0 & 0\\ 1 & 2 & 1 \end{array}\right],\;K_{135^{\circ }}=\left[\begin{array}{ccc} 0 & 1 & 2\\ -1 & 0 & 1\\ -2 & -1 & 0 \end{array}\right]$$

Each directional feature is obtained by convolving the input with the corresponding fixed Sobel kernel, producing four orientation-specific edge feature maps. Formally, the directional edge features are computed as

(8) $$F_{\theta }=E_{\mathrm{in}}*K_{\theta },\;\theta \in \left\{0^{\circ },45^{\circ },90^{\circ },135^{\circ }\right\}$$

where ∗ denotes the convolution operation. To further refine these features, each $F_{\theta }$ is passed through a learnable conv2d layer, allowing the network to adaptively enhance salient edge structures while suppressing irrelevant patterns.

The final output edge map $E_{\mathrm{out}}$ is obtained by selecting the maximum response across all orientations:

(9) $$E_{\mathrm{out}}=\max_{\theta }\left(conv2d\left(F_{\theta }\right)),\;\theta \in \left\{0^{\circ },45^{\circ },90^{\circ },135^{\circ }\right\}\right)$$

where $E_{\mathrm{out}}\in R^{1\times H\times W}$ is the final edge prior map. This max-pooling operation across orientations ensures that the most prominent edges dominate the final representation, thereby providing a robust structural prior that guides the attention mechanism toward critical regions. By explicitly modeling multi-directional edges, EFE enhances the sensitivity of EGAB to essential QR code structures, which is particularly beneficial for challenging restoration tasks such as deblurring and decoding under severe degradation.

### 3.2. Lightweight and Efficient Network (LENet)

**Figure 7:**
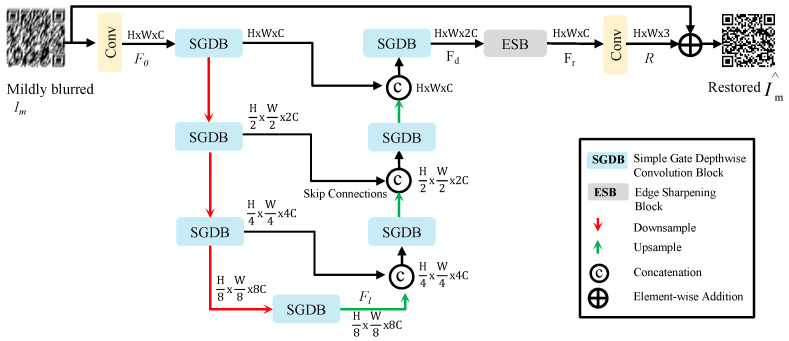
The overall architecture of LENet. We employ a U-shaped encoder–decoder architecture with multiple Simple Gate Depthwise Convolution Blocks (SGDB).

Similarly, LENet is implemented for fast deblurring of mildly blurred QR codes. As shown in Figure 7, it adopts a four-level U-shaped encoder–decoder architecture. The encoder consists of four Simple Gate Depthwise Convolution Blocks (SGDBs), and the decoder contains three, to efficiently extract and refine features. The decoder output is then passed to an Edge Sharpening Block (ESB), which employs a 3 × 3 depthwise convolution for spatial context refinement and edge enhancement.

#### 3.2.1. Simple Gate Depthwise Convolution Block (SGDB)

To efficiently restore mildly blurred QR codes, we design a lightweight Simple Gate Depthwise Convolution Block (SGDB) in the U-shaped encoder-decoder network. Inspired by efficient architectures such as MobileNetV2 [47] and NAFNet [17], SGDB follows a streamlined processing paradigm that decouples channel mixing and spatial aggregation while maintaining strong representational capacity.

**Figure 8:**
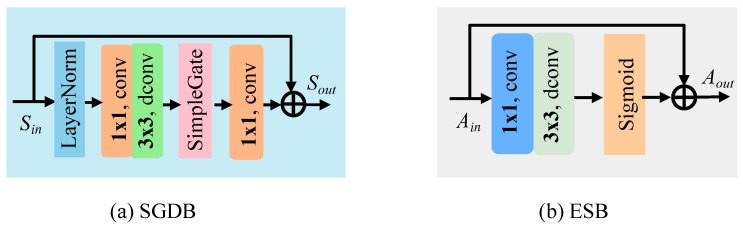
The core blocks of the LENet: (**a**) Simple Gate Depthwise Convolution Block (SGDB) that performs efficient feature extraction and refinement and (**b**) Edge Sharpening Block (ESB) that performs spatial context refinement and edge enhancement.

Figure 8a illustrates the processing pipeline of the input feature $S_{\mathrm{in}}$: layer normalization (LN), 1 × 1 convolution (Conv) for channel expansion, 3 × 3 depthwise convolution (dconv) for spatial context aggregation, SimpleGate (SG) [17], and another 1 × 1 convolution for channel contraction, with a weighted residual connection producing refined output $S_{\mathrm{out}}$. The overall operation can be formulated as

(10) $$S_{\mathrm{out}}=Conv\left(SG\left(Conv2d\left(Conv\left(LN\left(S_{\mathrm{in}}\right)\right)\right)\right)\right)+S_{\mathrm{in}}$$

This lightweight design efficiently combines channel mixing, spatial aggregation, and gated feature selection within a residual framework. Consequently, SGDB achieves a strong balance between efficiency and representational power, making it well-suited for lightweight QR code deblurring on mobile devices.

#### 3.2.2. Edge Sharpening Block (ESB)

To further enhance high-frequency details and refine structural edges in the reconstructed QR codes, we introduce an Edge Sharpening Block (ESB), which is appended after the output of the U-shaped encoder–decoder backbone, as shown in Figure 8b. ESB is employed to selectively emphasize edge-related features while preserving global content consistency through a lightweight residual formulation.

Given the intermediate feature map $A_{\mathrm{in}}\in R^{C\times H\times W}$ produced by the decoder, ESB generates a learned edge-aware attention map through a sequence of channel mixing, spatial context aggregation, and nonlinear gating operations. First, a 1×1 convolution is applied to $A_{\mathrm{in}}$ for channel mixing and feature fusion without altering spatial resolution. Next, the transformed features are processed by a 3×3 depthwise convolution for spatial context aggregation, which effectively enlarges the receptive field while maintaining computational efficiency. The resulting representation is passed through a sigmoid function to generate a soft spatial attention map where values are normalized into [0, 1] to indicate pixel-wise importance of edge-related regions. Finally, a residual connection is employed to preserve the fidelity of the reconstructed QR codes while selectively enhancing structural details. The overall operation of ESB is computed as

(11) $$A_{\mathrm{out}}=Sigmoid\left(Conv2d\left(Conv\left(A_{\mathrm{in}}\right)\right)\right)+A_{\mathrm{in}}$$

The proposed ESB is designed as a lightweight edge-aware refinement module. By generating an attention map that emphasizes edge-related features and applying it in a residual manner, ESB enhances important structural details without introducing significant computational overhead or causing over-sharpening. Consequently, it is well-suited for efficient deblurring of mildly blurred QR codes in LENet.

### 3.3. Blur Severity-Based Routing (BSR) Module

To achieve both a high decoding success rate and computational efficiency, we introduce the Blur Severity-based Routing (BSR) module, as illustrated in Figure 4. The BSR module categorizes a blurred input QR code $I_{b}$ as mildly blurred $I_{m}$ or severely blurred $I_{s}$ according to its Laplacian variance (LV). LV is used as a lightweight sharpness measure, where a higher value generally indicates sharper image content and a lower degree of blur.

The routing threshold τ was calibrated using only a subset sampled from the training data based on the decoding performance of LENet. Specifically, after applying LENet to the calibration samples, we calculated the LV values of the corresponding input images and divided them according to whether the LENet-restored outputs could be successfully decoded. The threshold was then defined as the midpoint between the minimum LV among the decoded samples and the maximum LV among the non-decoded samples:

(12) $$\tau =\frac{\min\left(L_{\mathrm{decodable}}\right)+\max\left(L_{\mathrm{non}-\mathrm{decodable}}\right)}{2}$$

where $L_{\mathrm{decodable}}$ and $L_{\mathrm{non}-\mathrm{decodable}}$ denote the set of LV values whose LENet-restored outputs are decodable and non-decodable, respectively. This calibration resulted in τ=100 in our experiments.

The algorithmic implementation of the BSR module is detailed in Algorithm 1.
**Algorithm 1** Blur Severity-Based Routing Strategy  1:**Input:** $I_{b}$: a blurred QR code,  2:    $M_{E}$: the large network (EG-Restormer).  3:    $M_{L}$: the lightweight network (LENet).  4:    τ: a pre-calibrated blur severity threshold.  5:    M(·): a blur metric function (Laplacian variance).  6:    D(·): a QR code decoder.  7:**Output:** Restored QR code $\hat{I}$.  8:**function**BlurRouter($I_{b},M_{E},M_{L},\tau ,M,D$)  9:      $lv\leftarrow M(I_{b})$                ▹ Calculate the blur severity score10:      **if** lv>τ **then**                      ▹ Case 1: mild blur detected11:            ${\hat{I}}_{L}\leftarrow M_{L}\left(I_{b}\right)$                                 ▹ Route to LENet12:            **if** $D({\hat{I}}_{L})$ succeeds **then**13:                  **return** ${\hat{I}}_{L}$14:            **else**15:                  ${\hat{I}}_{E}\leftarrow M_{E}\left(I_{b}\right)$             ▹ Fallback to EG-Restormer16:                  **return** ${\hat{I}}_{E}$17:            **end if**18:      **else**                                   ▹ Case 2: severe blur detected19:            ${\hat{I}}_{E}\leftarrow M_{E}\left(I_{b}\right)$                       ▹ Route to EG-Restormer20:            **return** ${\hat{I}}_{E}$21:      **end if**22:**end function**

During inference, an input with $\mathrm{LV}(I_{b})>\tau$ is regarded as mildly blurred and is first processed by LENet. Otherwise, it is classified as severely blurred and routed directly to EG-Restormer. If the output restored by LENet cannot be decoded, it is further redirected to EG-Restormer. This routing strategy avoids unnecessary computation for relatively mild cases while retaining stronger restoration capacity for severely blurred inputs.

## 4. Experiments

### 4.1. Datasets

We constructed a synthetic QR code Dataset (QRData), containing 1822 images degraded by non-uniform motion kernels for training and evaluation. To evaluate the effectiveness of the proposed QR code deblurring network in real-world scenarios, it is crucial to train and test the model on images that mimic physical camera motion. Conventional linear motion blur, which assumes constant velocity, often fails to capture the complex dynamics of handheld camera shake. Therefore, we propose a non-uniform motion blur synthesis algorithm that incorporates an acceleration parameter to simulate realistic trajectory dynamics.

(1) QR Code Image Generation

To construct QRData, we first generated a set of sharp QR code images with diverse structural configurations. The dataset contains QR codes with multiple versions, including V1–V7, V10–V12, V22, and V30. Thus, the selected versions cover both low-density QR codes with relatively large modules and high-density QR codes with more compact module arrangements.

The error-correction levels used in QRData include L and M. These two levels were selected because they are commonly used in practical applications where data capacity is important. Furthermore, compared with higher error-correction levels such as Q and H, levels L and M provide less redundancy, making the decoding process more sensitive to blur-induced structural degradation. Accordingly, they provide a more challenging setting for evaluating decoding-critical QR code deblurring.

(2) Non-uniform Motion Blur Synthesis

After generating the sharp QR code images, we synthesize motion-blurred QR codes using a non-uniform motion blur model. The degradation of a sharp QR code image I can be modeled as the convolution with a motion blur kernel (Point Spread Function, PSF) K, followed by the addition of noise N:

(13) B=I∗K+N

where B is the resulting blurred image. Unlike standard linear kernels, our kernel K is generated using a non-linear temporal mapping. Let *L* denote the total motion length and θ denote the motion angle. We define the normalized linear time as $t_{\mathrm{lin}}\in \left[0,1\right]$. To introduce non-uniformity, we apply a power-law transformation using an acceleration factor *a*:

(14) $$t_{\mathrm{acc}}={\left(t_{\mathrm{lin}}\right)}^{a}$$

where a>1 or a<1 correspond to different acceleration or deceleration profiles, respectively.

The spatial coordinates (x,y) of the motion trajectory within a kernel of size S×S are determined by

(15) $$\left\{\begin{array}{c} x\left(t_{\mathrm{acc}}\right)=t_{\mathrm{acc}}\cdot L\cos\left(\theta \right)-\frac{L\cos(\theta )}{2}+\frac{S}{2}\\ y\left(t_{\mathrm{acc}}\right)=t_{\mathrm{acc}}\cdot L\sin\left(\theta \right)-\frac{L\sin(\theta )}{2}+\frac{S}{2} \end{array}\right.$$

To ensure that the generated kernel is physically plausible, weights are assigned to each sampled point along the trajectory. The residence time at each coordinate determines its contribution to the PSF intensity. Bilinear interpolation is used to distribute the contribution of each continuous trajectory point to its four neighboring kernel pixels. Compared with nearest-neighbor assignment, this operation avoids abrupt and discontinuous kernel weights and produces a smoother discretization of the continuous motion path. Higher-order interpolation was not used because bilinear interpolation provides sufficient smoothness with low computational overhead and without introducing pronounced overshoot.

The complete non-uniform motion blur kernel synthesis procedure is summarized in Algorithm 2.
**Algorithm 2** Non-uniform Motion Blur Kernel Synthesis  1:**Input:** Length *L*, Angle θ, Acceleration factor *a*, Kernel size *S*  2:**Output:** Non-uniform motion blur kernel *K*  3:Initialize *K* as an S×S zero matrix  4:Sample $N_{p}$ points uniformly to create linear time vector $t_{\mathrm{lin}}=\left\{0,\ldots ,1\right\}$  5:Compute accelerated time vector: $t_{\mathrm{acc}}={\left(t_{\mathrm{lin}}\right)}^{a}$  6:**for** each $t\in t_{\mathrm{acc}}$ **do**  7:       $x\leftarrow t\cdot L\cos\left(\theta \right)-\frac{L\cos(\theta )}{2}+\frac{S}{2}$  8:       $y\leftarrow t\cdot L\sin\left(\theta \right)-\frac{L\sin(\theta )}{2}+\frac{S}{2}$  9:       Calculate weight *w* based on local velocity $\frac{d}{dt}\left(t_{\mathrm{acc}}\right)$10:       Perform *Bilinear Interpolation* to distribute weight *w* to 4-neighbor pixels in *K*11:**end for**12:Normalize *K* such that $\sum K_{i,j}=1$13:**return** *K*

**Figure 9:**
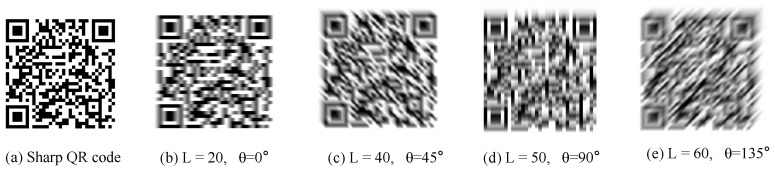
Example blurred QR codes from the proposed QRData dataset. (**a**) Sharp ground-truth QR code. (**b**–**e**) Synthesized non-uniform motion-blurred QR codes with different motion parameters: (**b**) mild horizontal blur (L = 20, θ = $0^{\circ }$), (**c**) medium diagonal blur (L = 40, θ = $45^{\circ }$), (**d**) severe vertical blur (L = 50, θ = $90^{\circ }$), and (**e**) severe opposite diagonal blur (L = 60, θ = $135^{\circ }$).

In the data generation pipeline, the kernel size *S* is set adaptively according to *L*. The motion length *L* is sampled from [20, 61] pixels, where *L* denotes the displacement extent of the point spread function in the image domain. Its effective severity depends on the spatial width of individual QR modules, which varies with the QR version and the proportion of the image occupied by the QR symbol. The angle θ is sampled from [0,π]. The acceleration exponent *a* controls the temporal non-uniformity of the motion trajectory. In QRData, *a* is sampled from [0.3, 0.7], corresponding to decelerating trajectories with non-uniform spatial exposure. This range was selected to introduce asymmetric blur while avoiding excessively concentrated PSFs. Although the formulation also permits (a>1) for accelerating trajectories, this regime is not included in the current dataset and is left for future investigation.This strategy effectively reduces the distributional discrepancy between synthetic and real camera motion blur compared with conventional linear-kernel synthesis. Representative samples from QRData are shown in Figure 9, illustrating the diversity of blur severity and motion directions produced by the proposed non-uniform motion blur synthesis algorithm.

(3) Quantitative Comparison with Real Camera Motion Blur

To quantitatively evaluate the similarity between synthetic and real camera motion blur, we compared three groups of motion-blurred QR codes: real images captured using a mobile camera, QR codes synthesized using the proposed non-uniform kernel with a∈[0.3,0.7], and QR codes synthesized using a conventional linear kernel with a=1. Edge Spread Functions (ESFs) were extracted from the finder-pattern boundaries, and the corresponding Line Spread Functions (LSFs) were used to calculate two blur descriptors: spatial skewness, which characterizes asymmetric edge spreading, and the 10–90% cumulative absolute-LSF energy width, which measures the effective extent of edge spreading. Distributional differences were evaluated using the Mann–Whitney U test with Holm correction, rank-biserial correlation as the effect size, and Wasserstein distance as a measure of distributional discrepancy.

As shown in Table 1, each synthetic group differed significantly from the real-camera group after Holm correction. Thus, neither synthesis strategy fully reproduced the distribution of real camera motion blur. Nevertheless, the proposed non-uniform model yielded consistently smaller Wasserstein distances than the conventional linear model for both LSF skewness (0.441 vs. 0.755) and transition width (5.654 vs. 6.899 pixels). This indicates that, in terms of the overall distributional discrepancy measured in the descriptor space, the proposed synthesis produced blur characteristics closer to those of real camera captures.

For LSF skewness, the proposed model also produced a smaller absolute rank-biserial correlation than the linear model (effect size magnitude = 0.500 vs. 0.750), indicating a reduced group separation. For transition width, however, the absolute rank–biserial correlation was larger for the proposed model (0.563 vs. 0.377), showing that the effect-size evidence was not uniformly favorable across all descriptors. Overall, the consistently reduced Wasserstein distances suggest that the proposed non-uniform synthesis provides an overall closer approximation to real motion-blur characteristics than conventional linear-kernel synthesis, although measurable differences remain.

(4) GoPro Dataset for Pre-training

In addition to the constructed QRData dataset, we also employ the GoPro dataset for training. The GoPro dataset contains 2103 training images and 1111 test images of various scenes with motion blur, providing a rich source of realistic blurred images for pre-training our models before fine-tuning on the QRData dataset. This two-stage training strategy enables the models to learn general deblurring features from the GoPro while adapting to the specific structural characteristics of QR code deblurring in QRData.

### 4.2. Metrics

Our models are trained using a two-stage transfer learning strategy: pre-training on the GoPro [48] dataset (2103 training images, 1111 test images), followed by fine-tuning on QRData (1521 training images, 201 validation images, 100 testing images). Moreover, we collected an independent real-world test set of 50 motion-blurred QR code images using the camera of a Realme smartphone. The QR codes covered Versions 1–6 and were captured at distances of approximately 5–10 cm. Motion blur was generated by moving the smartphone during image acquisition, with variations in motion direction and magnitude. The captured images also included variations in ambient illumination, viewing angle, and apparent QR code scale. These real-world images are not included in QRData and were not used for training, validation, or parameter calibration. Thus, the final evaluation consists of two disjoint subsets: 100 synthetic test images and 50 real-world captures.

Performance is evaluated using Peak Signal-to-Noise Ratio (PSNR), Structural SIMilarity (SSIM), and Decoding Rate (DR), defined as the percentage of successfully decoded QR codes. Model complexity is evaluated using parameter count and FLOPs (for a 512 × 512 image), while inference efficiency is evaluated by the Average inference time (Avg_time) per image in seconds.

### 4.3. Implementation Details

EG-Restormer is configured with [4, 6, 6, 8] Transformer blocks and [1, 2, 4, 8] attention heads across four levels, using 48 base channels [14]. All models are trained using the AdamW optimizer ($\beta_{1}$ = 0.9, $\beta_{2}$ = 0.999, weight decay = 0.0001). The initial learning rate is set to $3e^{-4}$ and gradually reduced to $1e^{-6}$ via cosine annealing. EG-Restormer employs progressive training for 400K iterations with the following (patch size, batch size) pairs: [($128^{2}$, 64), ($160^{2}$, 40), ($192^{2}$, 32), ($256^{2}$, 16), ($320^{2}$, 8), ($384^{2}$, 8)]. LENet is trained for 1000K iterations with a fixed patch size of 256 × 256 and a batch size of 8. For data augmentation, we use flipping, rotation, and shuffling. For the decoder of ADNet, the Zbar library is used for all decoding attempts.

All experiments are conducted on a system running Ubuntu 20.04.6 LTS, with Python 3.8.2 and PyTorch 2.3.1. The hardware platform consists of an Intel(R) Xeon(R) Gold 6226R CPU @ 2.90 GHz and four NVIDIA RTX A6000 GPUs. To ensure a fair comparison, all models are implemented in PyTorch and trained under the same hardware configuration.

### 4.4. Results

We compare EG-Restormer with two representative general-purpose image-deblurring methods, including NAFNet-32 [17] and Restormer [14]. Quantitative results for two training strategies are reported in Table 2 and Table 3.

**Pre-training on GoPro only.** We first evaluate the quantitative performance of models pre-trained exclusively on the GoPro dataset. EG-Restormer is compared with two representative general-purpose image-deblurring methods, NAFNet-32 and Restormer, as shown in Table 2. Among all methods, EG-Restormer obtains the best performance across all three metrics: with a PSNR of 10.99 dB, which is 0.39 dB higher than Restormer and 0.79 dB higher than NAFNet-32; an SSIM of 0.582, which is 0.086 higher than Restormer and 0.133 higher than NAFNet-32; and a DR of 58.67%, which is 8.67% higher than Restormer and 18.00% higher than NAFNet-32. These results demonstrate that, even without QR-specific training data, the explicit edge guidance mechanism of EG-Restormer effectively restores structural integrity under severe motion-blur conditions, leading to improved decodability.

**Fine-tuning on QRData.** After fine-tuning on the synthetic QRData training set, all methods exhibit substantial performance improvements, as reported in Table 3. Restormer achieves the best reconstruction fidelity, with a PSNR of 18.15 dB and SSIM of 0.766. In contrast, EG-Restormer attains a decoding rate of 90.00%, outperforming Restormer, NAFNet-32, Dong et al. [21], and Gu et al. [23] by 1.33, 8.67, 10.00, and 27.33 percentage points, respectively. Notably, EG-Restormer achieves a slightly higher decoding rate than Restormer after fine-tuning, indicating that explicitly incorporating QR-specific edge priors is beneficial for preserving structures required for successful decoding.

**Separate Evaluation on the Synthetic and Real-world Test Sets.** To further evaluate the generalization ability of the proposed method, we separately report the results on the synthetic QRData test subset and the independently captured real-world subset.

As shown in Table 4, after fine-tuning on QRData, all methods achieve 100% decoding accuracy on the synthetic subset, indicating that the synthetic training data are well learned. Meanwhile, the real-world subset remains considerably more challenging due to practical imaging variations. EG-Restormer obtains the highest decoding rate of 70% on the real-world subset, outperforming Restormer by 4 percentage points and NAFNet-32 by 26 percentage points, demonstrating the effectiveness of incorporating explicit edge priors for practical QR code restoration.

**Analysis of Fidelity Metrics and Decoding Success.** The discrepancy between fidelity metrics and decoding rate may be explained by the different reconstruction biases introduced by the two architectures. Both Restormer and EG-Restormer are optimized using image-restoration supervision without direct decoder feedback. However, PSNR and SSIM measure global reconstruction fidelity and do not distinguish errors in decoding-critical regions from those in less important areas. QR decoding depends strongly on the integrity of finder patterns, timing patterns, alignment patterns, and local black–white module transitions. Consequently, even a small number of localized structural errors may cause decoding failure while contributing only modestly to the global fidelity scores. EG-Restormer incorporates multi-directional edge priors through EGAB, introducing an architectural bias toward decoding-critical boundaries during feature aggregation. This bias may improve QR readability even when local intensity deviations or edge-enhancement artifacts reduce the overall PSNR and SSIM.

**Figure 10:**
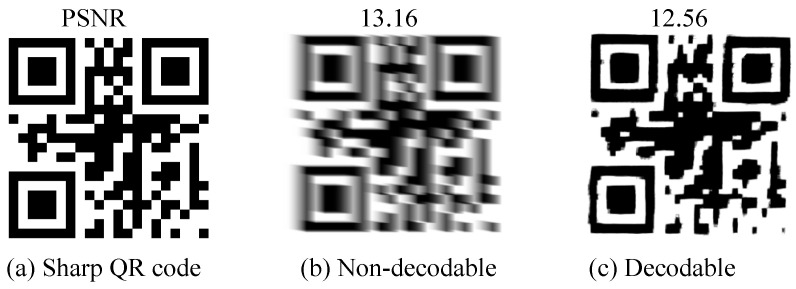
Representative examples showing that higher PSNR does not necessarily imply successful QR decoding. (**a**) Sharp reference QR code. (**b**) A restored QR image with a higher PSNR of 13.16 dB that remains undecodable. (**c**) A restored QR image with a lower PSNR of 12.56 dB that can still be successfully decoded.

Representative cases illustrating this mismatch are shown in Figure 10. The restored image in Figure 10b achieves a slightly higher PSNR of 13.16 dB but remains undecodable, indicating that its residual degradation affects structures required for localization or module sampling. Conversely, the image in Figure 10c has a slightly lower PSNR of 12.56 dB but remains decodable because its essential functional patterns and module topology remain sufficiently recognizable.

These results indicate that PSNR and SSIM should be regarded as complementary rather than sufficient metrics for QR code restoration. They quantify global image fidelity, whereas decoding rate directly reflects whether the recovered structural information supports downstream QR recognition. Hence, the evaluation of QR code deblurring methods should jointly consider pixel-level fidelity, structural preservation, and decoding performance.

**Figure 11:**
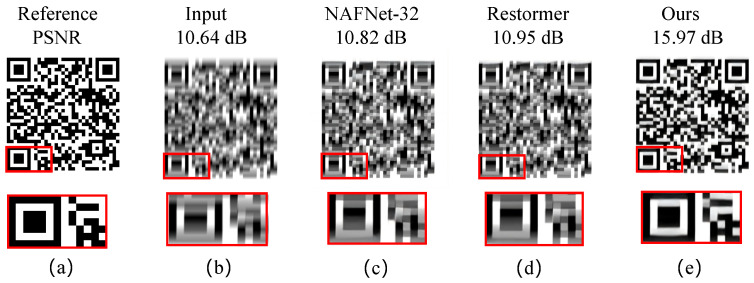
Qualitative comparison of QR code deblurring on the test set. (**a**) Original sharp; (**b**) blurred input; (**c**) NAFNet-32 output; (**d**) Restormer output; (**e**) EG-Restormer (ours).

**Qualitative analysis.** To validate the effectiveness of our EG-Restormer, Figure 11 presents a qualitative comparison among EG-Restormer, Restormer, and NAFNet on severely motion-blurred QR codes. The input blur severely smears edges, disrupts the finder patterns, and makes individual modules difficult to recognize. After deblurring, NAFNet fails to recover fine structures and produces discontinuous stripes that cannot be recognized by the decoder. Restormer partially removes the blur but still leaves visible trailing artifacts and blurred boundaries; as a result, the restored QR code remains undecodable. In contrast, EG-Restormer reconstructs sharper and more continuous structural edges, restores the integrity of all three finder patterns, and preserves clear module separations, producing a clean and successfully decodable QR code. This qualitative result confirms that explicit edge priors are important for achieving successful decoding under severe non-uniform motion blur, whereas pixel-wise accuracy alone is insufficient.

**Complexity analysis.** Table 5 reports the model complexity and runtime of the compared methods. LENet, our lightweight baseline, requires only 0.28 M parameters and 3.87 G FLOPs, with an average inference time of 0.23 s per image, which is substantially lower than those of the other methods. This makes LENet suitable for latency-sensitive QR code deblurring scenarios, although at the cost of lower decodability, with a DR of 49.33% after joint training, as shown in Table 3. Among the high-performance models, NAFNet-32 contains 17.11 M parameters and 64.44 G FLOPs, achieving an average inference time of 0.794 s. Restormer and our EG-Restormer have similar parameter counts (≈26.1 M) and FLOPs (≈565 G), with EG-Restormer introducing only a slight overhead (+0.15 G FLOPs and +0.072 s) due to its explicit edge guidance modules. Despite this modest increase in complexity, EG-Restormer attains the highest decoding rate (90.00%), suggesting that the additional computational cost is justified for QR code deblurring tasks, where decoding success is the primary objective.

### 4.5. Ablation Study

#### 4.5.1. Effect of the Edge-Modulation Coefficient α

The coefficient α controls the strength of the edge prior injected into the query and key representations of EGAB. To evaluate its influence, we tested α∈{0,0.1,0.2,0.3,0.4,0.5} under the same training and evaluation settings. The case α=0 disables the explicit edge modulation and therefore serves as a reference for assessing the contribution of the edge-guided term.

As shown in Table 6, increasing α from zero initially improves the decoding rate, indicating that moderate edge guidance helps preserve decoding-critical module boundaries and functional patterns. However, an excessively large α may overemphasize local edge responses, disturb the balance between structural and contextual features, and introduce edge-enhancement artifacts, thereby reducing restoration fidelity or decoding performance. The highest decoding rate is obtained at α=0.1, which is used in all subsequent experiments.

#### 4.5.2. Ablation of the Adaptive Routing Strategy

To evaluate whether the proposed BSR module provides an effective accuracy–efficiency trade-off, we compare ADNet with four inference configurations: Restormer, EG-Restormer, LENet, and ADNet with random branch selection. EG-Restormer and LENet represent the two fixed-branch strategies, whereas ADNet-Random evaluates whether the improvement results from blur-aware routing rather than simply combining the two restoration networks. Decoding rate (DR) and average inference time are reported to evaluate decoding performance and computational efficiency, respectively.

As shown in Table 7, EG-Restormer achieves the highest decoding rate but also requires the longest average inference time, whereas LENet provides substantially faster inference at the cost of a considerable reduction in decoding performance. Randomly selecting a restoration branch reduces the average runtime but achieves a DR of only 72.00%, indicating that simply combining the two branches is insufficient. By routing inputs according to their estimated blur severity and applying the fallback mechanism when LENet restoration remains undecodable, ADNet achieves the same DR of 90.00% as EG-Restormer while reducing the average inference time from 0.910 s to 0.737 s, corresponding to a 19.0% reduction. Compared with ADNet-Random, the proposed routing strategy improves DR by 18.00 percentage points. These results validate the effectiveness of blur-aware routing in balancing decoding performance and average inference efficiency.

#### 4.5.3. Sensitivity Analysis of the Routing Threshold τ

To evaluate the sensitivity of the routing threshold, we varied the routing threshold τ while keeping all network parameters unchanged.

As shown in Table 8, increasing τ routes a larger proportion of inputs directly to EG-Restormer, while reducing the percentage of images that are forwarded through the fallback mechanism after LENet restoration. Consequently, the average inference time gradually increases because more images are processed by the computationally intensive restoration branch in the first stage. Since ADNet incorporates a decoding-failure fallback mechanism, the routing threshold primarily affects the execution path and computational cost rather than the final decoding capability. The threshold τ=100, which is automatically determined from the training subset using the midpoint calibration rule, provides a reasonable operating point under the current experimental setting.

## 5. Discussion

The results reveal two main observations. First, explicit edge guidance is particularly useful when the model is transferred from natural-image deblurring to QR restoration. Second, fidelity metrics and decoding performance are not fully aligned because decoding depends on localized functional patterns and module decisions. These findings motivate the following discussion of the current limitations and deployment prospects.

### 5.1. Limitations and Improvement Directions

The current BSR module uses a fixed Laplacian-variance threshold τ=100, calibrated on the training subset. Because Laplacian variance is also affected by resolution, QR module size, illumination, contrast, sensor noise, and in-camera processing, this value should be regarded as an empirical operating point rather than a universally optimal threshold. A feasible improvement is to replace it with a lightweight router that predicts branch-selection probabilities from normalized blur, contrast, noise, and module-scale features. Such a router could be jointly optimized using a loss that balances restoration or decoding performance against computational cost.

The current synthetic blur model includes only temporal exponents a<1. A more complete synthesis framework should include a<1, a=1, and a>1 and report sensitivity to the acceleration regime. More realistic motion could also be generated using piecewise acceleration, curved trajectories, or camera-motion traces obtained from inertial sensors.

Performance on extremely dense QR codes depends strongly on the number of pixels per module. When the module width is only a few pixels and the blur extent exceeds several module widths, neighboring modules become strongly mixed. A possible solution is a scale-adaptive pipeline that estimates pixels per module and selects an appropriate restoration resolution or network branch. Module-aware losses and targeted fine-tuning on dense QR versions could further improve structural recovery.

Beyond QR codes, the edge-guided attention mechanism may be applicable to other structured binary data representations, including ASCII-inspired and Bose–Chaudhuri–Hocquenghem (BCH)-based optical data containers. However, the complete ADNet framework remains QR-specific because its training data, routing criterion, decoder, and fallback mechanism are designed for QR images. Transfer to another binary representation would require task-specific training data, router recalibration, and replacement of the QR decoder.

The current real-world evaluation includes only 50 QR code images captured using a single smartphone and should therefore be regarded as a preliminary validation rather than comprehensive evidence of deployment robustness. Although the dataset covers several imaging variations, future work will construct a larger multi-device, multi-condition benchmark for more comprehensive evaluation under practical deployment scenarios.

### 5.2. Deployment Prospects

The complexity and runtime results in Table 5 indicate that adaptive routing is more resource-efficient than applying EG-Restormer to every input. LENet processes mildly blurred samples with lower computational cost, whereas EG-Restormer is invoked only for severely blurred or decoding-failed cases. This strategy reduces average inference latency by 19% while maintaining comparable decoding performance.

For fully on-device deployment, QR localization, BSR, LENet, decoding, and EG-Restormer can be integrated into a unified pipeline. Processing only the detected QR region, together with reduced-precision inference, quantization, structured pruning, knowledge distillation, operator fusion, and mobile Neural Processing Unit (NPU) optimization, could further reduce memory use and latency. A compressed EG-Restormer branch could also be developed by reducing feature dimensions, Transformer blocks, or attention heads.

Nevertheless, the current runtime was measured on the experimental hardware used in this study and does not directly demonstrate real-time execution on a low-power mobile processor. Future work should benchmark the complete pipeline on representative mobile systems-on-chip and report latency, memory consumption, energy usage, routing proportions, and fallback frequency.

## 6. Conclusions

In this paper, we proposed a domain-specific framework for QR code motion deblurring by directly integrating edge priors into a Transformer-based network. The proposed Edge-Guided Attention Block (EGAB) enables the network to focus on restoring the sharp edges that are crucial for QR code recognition. Furthermore, the Adaptive Dual-network framework, ADNet, combines a lightweight network with a more powerful restoration network, reducing average inference latency without sacrificing performance. Comprehensive experiments demonstrate that the proposed method achieves a slightly higher decoding rate than Restormer after fine-tuning. This work provides a promising direction for adapting general vision models to specialized tasks with strong structural priors, potentially benefiting related tasks such as text images, barcodes, and other structured binary visual patterns. The proposed method shows potential for latency-sensitive QR scanning applications. Future research could explore more sophisticated routing strategies, larger and more diverse datasets, and extensions to other types of structured visual data.

## Figures and Tables

**Table 1:** Distributional comparisons between real and synthetic motion blur. Holm-corrected Mann-Whitney U *p*-values, rank-biserial effect sizes, and Wasserstein distances are reported.

Metric	Comparison	*U*	Corrected *p*	Effect Size	Wasserstein
LSF skewness	Ours vs. Real	150.0	0.006	−0.500	0.441
LSF skewness	Linear vs. Real	75.0	<0.001	−0.750	0.755
Transition width	Ours vs. Real	469.0	0.003	0.563	5.654
Transition width	Linear vs. Real	413.0	0.026	0.377	6.899

**Table 2:** Comparison of deblurring methods trained on GoPro (evaluated on the combined test set consisting of 100 synthetic QRData images and 50 real-world captures).

Methods	GoPro		
	DR (%)	PSNR (dB)	SSIM
LENet (Ours)	32.67	9.78	0.421
NAFNet-32 [17]	40.67	10.20	0.449
Restormer [14]	50.00	10.60	0.496
EG-Restormer (Ours)	**58.67**	**10.99**	**0.582**

**Table 3:** Comparison of deblurring methods trained on GoPro + QRData (evaluated on the combined test set consisting of 100 synthetic QRData images and 50 real-world captures).

Methods	GoPro + QRData		
	DR (%)	PSNR (dB)	SSIM
LENet (Ours)	49.33	11.37	0.572
NAFNet-32 [17]	81.33	15.56	0.695
Restormer [14]	88.67	**18.15**	**0.766**
Dong et al. [21]	80.00	16.55	0.760
Gu et al. [23]	62.67	13.07	0.69
EG-Restormer (Ours)	**90.00**	14.88	0.666

**Table 4:** Separate evaluationon the synthetic QRData test subset and the captured real-world subset.

Training	Methods	Synthetic QRData			Real-World		
		PSNR	SSIM	DR (%)	PSNR	SSIM	DR (%)
GoPro	NAFNet-32	11.04	0.485	49	8.51	0.376	24
	Restormer	11.60	0.550	59	8.58	0.389	32
	EG-Restormer	**12.16**	**0.596**	**68**	**8.65**	**0.393**	**40**
GoPro + QRData	NAFNet-32	21.10	0.829	100	8.47	0.370	44
	Restormer	**22.03**	**0.938**	100	8.39	**0.421**	66
	EG-Restormer	18.06	0.802	100	**8.52**	0.395	**70**

**Table 5:** Comparison of model complexity and runtime for different deblurring methods.

Methods	Params. (M)	Flops (G)	Avg_Time (s)
LENet (Ours)	**0.28**	**3.87**	**0.23**
NAFNet-32 [17]	17.11	64.44	0.794
Restormer [14]	26.13	564.96	0.838
EG-Restormer (Ours)	26.13	565.14	0.910

**Table 6:** Sensitivity of EG-Restormer to the edge-modulation coefficient α.

α	DR (%)	PSNR (dB)	SSIM
0.0	88.67	18.15	0.766
0.1	90.00	14.88	0.666
0.2	69.33	14.53	0.698
0.3	68.67	15.91	0.730
0.4	67.33	16.47	0.729
0.5	65.33	15.74	0.726

**Table 7:** Ablation study of the blur severity-based routing strategy.

Methods	Avg_Time (s)	DR (%)
Restormer (baseline)	0.838	88.67
EG-Restormer	0.910	**90.00**
LENet	**0.28**	49.33
ADNet (random)	0.457	72.00
ADNet (ours)	0.737	**90.00**

**Table 8:** Effect of the routing threshold τ on routing proportions and inference time.

τ	Direct EG-Restormer (%)	Fallback (%)	Avg_Time (s)
20	1.33	62.00	0.672
50	18.00	48.67	0.682
80	31.33	39.33	0.700
100	44.67	32.00	0.737
120	54.00	26.00	0.763
150	55.33	24.67	0.764

## Data Availability

The source code, trained models, and the 50-image real-world evaluation subset will be made publicly available upon acceptance at https://github.com/leejianping/ADNet (accessed on 14 July 2026).
